# P-796. Using Interruptive Alerts for Diagnostic Stewardship of the BioFire Pneumonia PCR Panel

**DOI:** 10.1093/ofid/ofaf695.1006

**Published:** 2026-01-11

**Authors:** Alexander S Plattner, Valerie Yuenger, Christine R Lockowitz, Monica Abdelnour, Vahid Azimi, Stephanie Bledsoe, Rebekah Dumm, Evan E Facer, Nicholas B Hampton, Ronald Jackups, Elizabeth Neuner, Jason G Newland, Matthew M Sattler, Nasiri Sarawanangkoor, Sena Sayood, Melanie L Yarbrough, Rebecca Same

**Affiliations:** WashU Medicine / St. Louis Children's Hospital, St Louis, MO; St. Louis Children's Hospital, St. Louis, Missouri; St. Louis Children’s Hospital, Trenton, Illinois; Washington University, St. Louis, Missouri; Washington University School of Medicine, Oakland, California; BJC HealthCare, St Louis, Missouri; Washington University School of Medicine, Oakland, California; St. Louis Children's Hospital, St. Louis, Missouri; BJC Healthcare, St. Louis, Missouri; Washington University in St Louis, St Louis, Missouri; Barnes-Jewish Hospital, St. Louis, Missouri; Nationwide Children's Hospital, Columbus, OH; Washington University in St. Louis School of Medicine, St. Louis, MO; Washington University in St Louis, St Louis, Missouri; Washington University School of Medicine, Oakland, California; Washington University School of Medicine in St. Louis, St. Louis, Missouri; Children's Hospital of Philadelphia, Philadelphia, PA

## Abstract

**Background:**

The BioFire Pneumonia Panel (BFPP) uses multiplex polymerase chain reaction technology to detect pneumonia-causing pathogens and select antimicrobial resistance genes. While initial studies suggested one possible benefit of the BFPP was to aid in promptly narrowing antibiotics, evaluation of real-world use shows little impact on antibiotic use, especially for repeat tests during the same admission and tests from tracheostomies.

**Figure 1.**
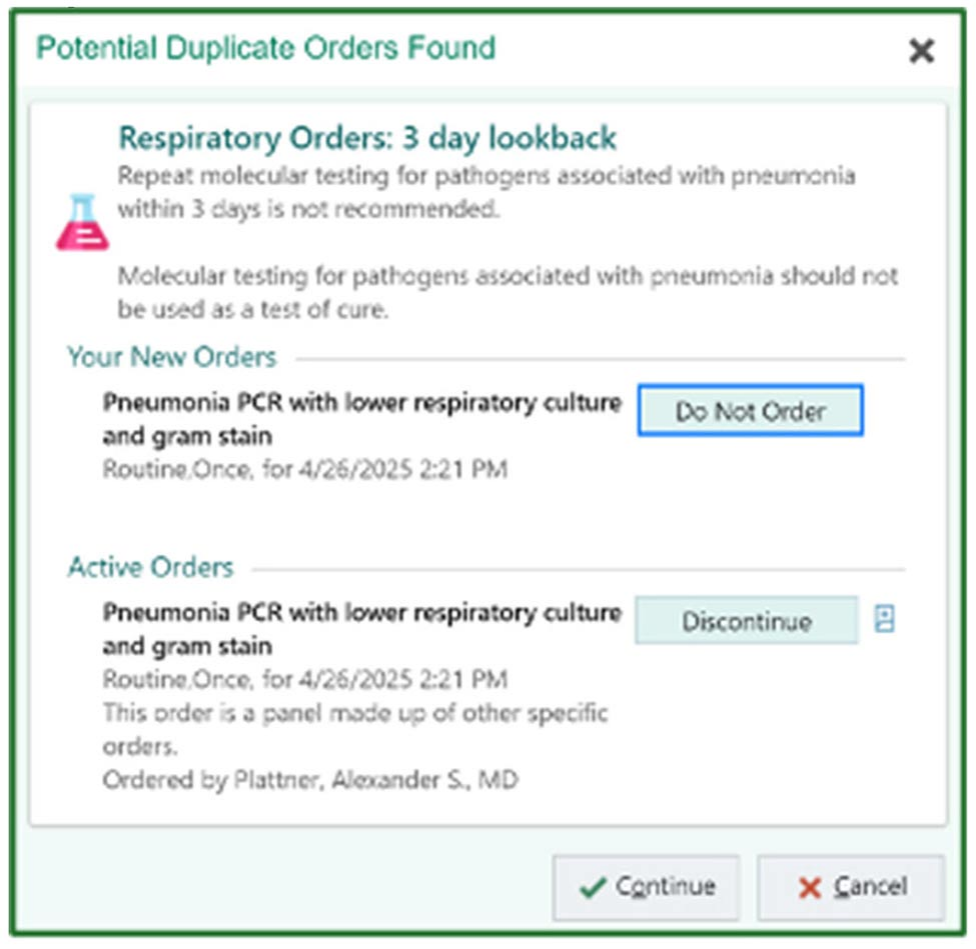
Duplicate Order Alert Interruptive alert for attempts to order a repeat BFPP within 72 hours of a prior BFPP. Clinician choices of “Do Not Order” or “Cancel” were considered successful interventions.

**Figure 2.**
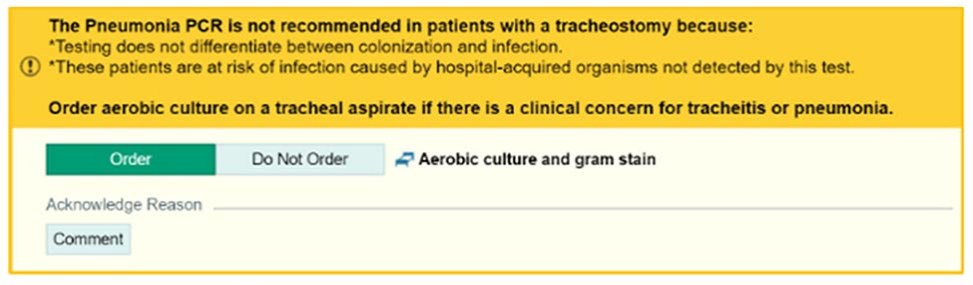
Tracheostomy Alert. Interruptive alert for attempts to order a BFPP on a patient with a tracheostomy in place for more than 72 hours. Clinicians choosing to order the aerobic culture and gram stain instead of the BFPP was considered a success.

**Methods:**

In response to high institutional usage, clinical decision support (CDS) tools were developed to decrease BFPP use in low yield clinical scenarios. Two interruptive alerts were built into our electronic health record (EHR; Epic Systems) to fire upon ordering BFPP for patients of all ages. Beginning September 2021, a duplicate order alert fired if a BFPP or respiratory viral panel had been reported in the prior 72 hours (Fig 1). Beginning late January 2024, a second alert fired for patients with tracheostomies in place longer than 72 hours, recommending routine respiratory culture instead (Fig 2). Alert performance and BFPP usage were evaluated using EHR reporting tools. Alert firing was a “success” if the clinician did not proceed with the BFPP order.

**Figure 3.**
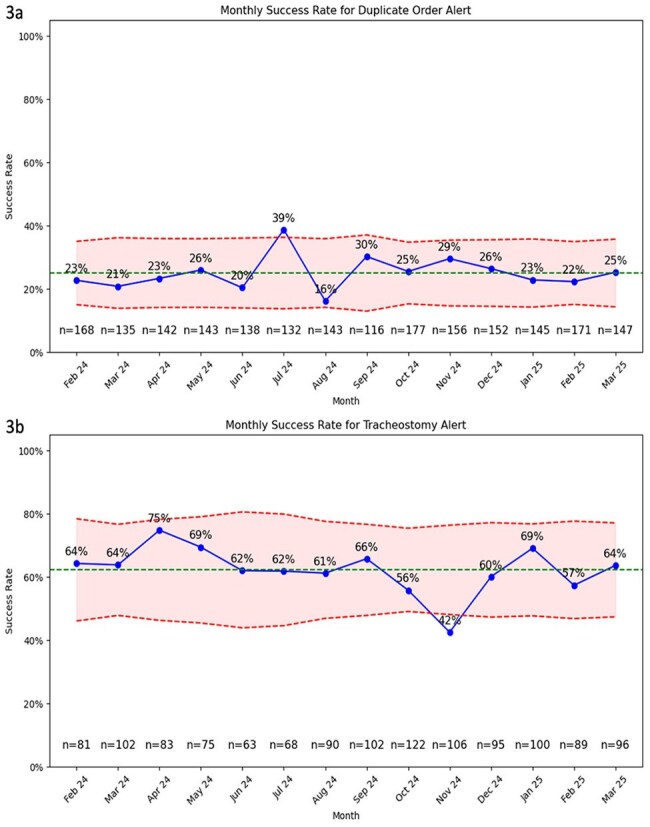
P-charts of Monthly Alert Success P-charts showing performance of the duplicate order alert (3a) and tracheostomy alert (3b). Monthly success rate (blue) is shown with upper and lower control limits (red) surrounding the median line (green). Total monthly alerts are indicated along the x-axis.

**Figure 4.**
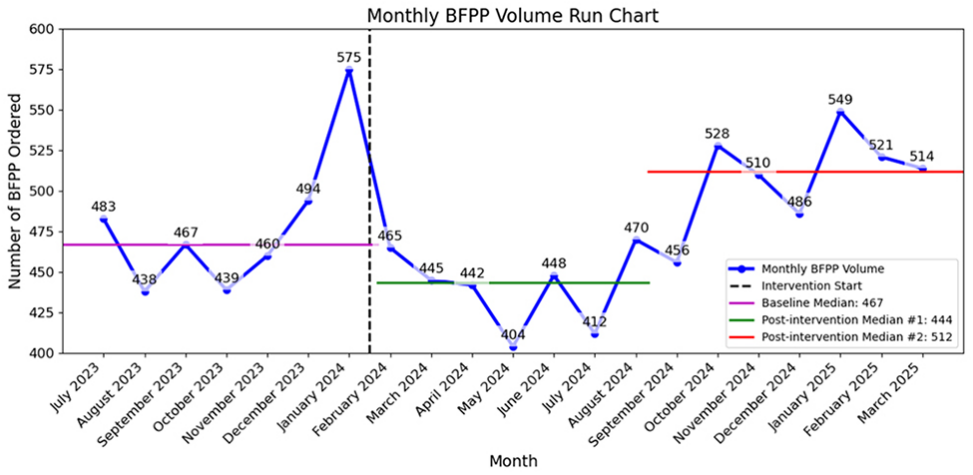
Run Chart of Monthly BFPP Tests Performed Run chart showing total monthly BFPP tests obtained across seven hospitals serviced by the Barnes Jewish Hospital Microbiology Lab. The date of tracheostomy alert introduction is marked by the intervention start.

**Results:**

Monthly performance of the two alerts is shown as p-charts (Fig 3). Starting February 2024, the repeat test alert fired 2,065 times over 14 months (148 per month), resulting in 512 new tests avoided (25%); however, 49 were followed by an order later that day (10%). The tracheostomy alert fired 1,183 times (85 per month), resulting in 728 tests avoided (62%), with 232 followed by an order later that day (32%). While there was a significant reduction in BFPP ordering after the second alert's introduction; overall ordering has now surpassed baseline (Fig 4).

**Conclusion:**

Interruptive alerts reduced 959 unnecessary BFPP orders, but further stewardship is needed. The alerts’ success ranged from 25% (duplicate order alert) to 62% (tracheostomy alert) and there was an initial decline in monthly BFPP orders following introduction of the tracheostomy alert. However, these improvements were not sustained and BFPP ordering eventually exceeded baseline levels. Future efforts are required for CDS refinement and root cause analysis to evaluate the increase in test ordering.

**Disclosures:**

Christine R. Lockowitz, PharmD, BCIDP, AbbVie: Grant/Research Support|Premier Inc.: Honoraria Rebekah Dumm, PhD D(ABMM), BD: Advisor/Consultant|BD: Grant/Research Support|Biomerieux: Advisor/Consultant|Biomerieux: Grant/Research Support|Diasorin: Grant/Research Support|Pattern Biosciences: Grant/Research Support|Qiagen: Grant/Research Support|Roche Diagnostics: Advisor/Consultant|Shionogi: Advisor/Consultant Evan E. Facer, DO, AbbVie, Inc.: Grant/Research Support Ronald Jackups, Jr., MD, PhD, Siemens: Contracted research|Werfen: Advisor/Consultant|Werfen: Contracted research Jason G. Newland, MD, MEd, Pfizer: Grant/Research Support Melanie L. Yarbrough, PhD, Becton Dickinson: Advisor/Consultant|bioMerieux: Advisor/Consultant|bioMerieux: Grant/Research Support|Shionogi: Advisor/Consultant

